# Both ERK1 and ERK2 Are Required for Enterovirus 71 (EV71) Efficient Replication

**DOI:** 10.3390/v7031344

**Published:** 2015-03-20

**Authors:** Meng Zhu, Hao Duan, Meng Gao, Hao Zhang, Yihong Peng

**Affiliations:** Department of Microbiology, School of Basic Medical Sciences, Peking University Health Science Center, 38 Xueyuan Road, Beijing 100191, China; E-Mails: mengzhu1984@gmail.com (M.Z.); dight119@163.com (H.D.); gm0323@126.com (M.G.); hao_zhang@bjmu.edu.cn (H.Z.)

**Keywords:** EV71, virus replication, ERK1, ERK2

## Abstract

It has been demonstrated that MEK1, one of the two MEK isoforms in Raf-MEK-ERK1/2 pathway, is essential for successful EV71 propagation. However, the distinct function of ERK1 and ERK2 isoforms, the downstream kinases of MEKs, remains unclear in EV71 replication. In this study, specific ERK siRNAs and selective inhibitor U0126 were applied. Silencing specific ERK did not significantly impact on the EV71-caused biphasic activation of the other ERK isoform, suggesting the EV71-induced activations of ERK1 and ERK2 were non-discriminative and independent to one another. Knockdown of either ERK1 or ERK2 markedly impaired progeny EV71 propagation (both by more than 90%), progeny viral RNA amplification (either by about 30% to 40%) and protein synthesis (both by around 70%), indicating both ERK1 and ERK2 were critical and not interchangeable to EV71 propagation. Moreover, suppression of EV71 replication by inhibiting both early and late phases of ERK1/2 activation showed no significant difference from that of only blocking the late phase, supporting the late phase activation was more importantly responsible for EV71 life cycle. Taken together, this study for the first time identified both ERK1 and ERK2 were required for EV71 efficient replication and further verified the important role of MEK1-ERK1/2 in EV71 replication.

## 1. Introduction

Enterovirus 71 (EV71) is a non-enveloped, positive single-stranded RNA virus belonging to the enterovirus A species of the genus *Enterovirus*, family *Picornaviridae* [1]. The EV71 genome, about 7.5 kb in length, consists of a single open reading frame encoding seven nonstructural proteins (2A, 2B, 2C, 3A, 3B, 3C, and 3D) and four structural proteins (VP1, VP2, VP3, and VP4) [2]. As one of the major causative agents leading to large outbreaks of hand, foot and mouth disease (HFMD) worldwide, especially in the Asia-Pacific area recently, EV71 has become the most dangerous neurotropic enterovirus after the control of poliovirus [3,4]. There is currently no effective vaccine or specific therapy that has been applied to prevent or treat EV71-caused severe HFMD, due to insufficient understanding of the molecular mechanisms of EV71 replication and host response to EV71 infection.

It is universally acknowledged that successful viral replication is reliant on many functioning components of cellular metabolism and a prerequisite for all the following pathogenic consequences in host cells. Accumulated data show that virus takes advantage of various signaling cascades for its life cycle, among which studies done by us and others have demonstrated that the extracellular signal regulated kinase (ERK) pathway is essential for EV71 and other viruses replication, inhibition of this signaling pathway has been found to severely impair EV71 and other variety of viruses production [5,6,7,8,9,10,11].

Cellular ERK signaling pathway, one of the three major mitogen-activated protein kinase (MAPK) cascades, which consists of three tiered serine/threonine kinases of Raf, MEK and ERK, plays an important role in regulating cell physiological functions [12,13,14] as well as many pathologic processes, including brain injury, cancer, diabetes, infectious diseases and inflammation *etc.* [15,16,17,18]. The two isoforms of ERKs, ERK1 and ERK2 (also referring to ERK1/2), are considered to be the only downstream substrates of MEK (including MEK1 and MEK2, also referring to MEK1/2) to date [19]. Therefore, ERK1 and ERK2, undertaking the upstream signals from MEK1/2 and in turn activating variety of their downstream substrates, are key players in ERK pathway. They share 85% similarity at the amino acid level [20] and yet it is still controversial whether the individual ERK isoform plays a distinctive role(s). Some studies suggest that ERK1 and ERK2 are interchangeable [21,22,23]. However, considerable evidences indicate that they might act differentially [24,25,26,27]. Thus, it is still an open question, which needs to be further explored as to whether roles are unique or preferred to one or the other ERK isoform in the physiological and/or pathological processes.

Our previous work have proved that MEK1 and MEK2 play differential roles, and MEK1, rather than MEK2, is critical to promote EV71 efficient replication [6], highlighting that MEK1 and MEK2 could exert distinct effects on the replication of EV71. However, as the downstream kinases of MEK1, the specific contributions of ERK1 and ERK2 to EV71 replication have not been addressed yet. The objective of the present study is to determine the role(s) of individual ERK isoform on the life cycle of EV71. In addition, here we showed that either ERK1 or ERK2 were both required and not functionally redundant for EV71 efficient replication.

## 2. Materials and Methods

### 2.1. Cells Culture and Virus Preparation

Rhabdomyosarcoma (RD) cell line was obtained from The National Institute for the Control of Pharmaceutical and Biological Products. Cells were cultured in Dulbecco’s modified Eagle’s medium (DMEM, GIBCO) supplemented with 10% fetal bovine serum (FBS, Gibco) at 37 °C in an atmosphere of 5% CO_2_.

The titer of Enterovirus 71 (EV71-BC08 stain) was determined by titration in RD cells and stored at −80 °C until use [28].

### 2.2. Inhibitor against ERK Pathway and Antibodies

U0126 (Pierce, Thermo Scientific, Waltham, MA, USA), the inhibitor of ERK pathway, was dissolved in DMSO at the stocking concentration of 2 mM. Antibodies were purchased from Cell Signaling Technology (Danvers, MA, USA) (CST, anti-ERK1/2, anti-phospho-ERK1/2), Abcam (anti-EV71 VP1 and anti-VP3/4), Santa Cruz (anti-β-actin).

### 2.3. siRNAs and Transfection

siRNAs targeting human ERK1 (siERK1) and ERK2 (siERK2) were synthesized from Genepharma Co., Ltd. (Shanghai, China). The sequences, coming from Christopher A. Dimitri’s paper [29], are showing as follows: 

ERK1 siRNA (siERK1): 5'-CCCUGACCCGUCUAAUAUAdTdT-3' (sense), 

5'-UAUAUUAGACGGGUCAGGGdAdG-3' (antisense); 

ERK2 siRNA (siERK2): 5'-CAUGGUAGUCACUAACAUAdTdT-3'(sense),

5'-UAUGUUAGUGACUACCAUGdAdT-3' (antisense).

In addition, the negative control siRNAs (siNC) were purchased from Genepharma Co., Ltd. siERK1 and siERK2 (siERK1+2) were used together to knock down both ERK1 and ERK2.

Lipofectamine 2000 (Invitrogen) was used according to the manufacturer’s instructions for siRNA transfection. RD cells were grown to 60% confluency in 6 or 12 well plates before transfection. RD cells were then transfected with siRNAs at the indicated concentrations.

### 2.4. Morphological Analysis

RD cells infected with EV71 were examined at every 8 h intervals post infection (p.i.) for the cytopathic effect (CPE) with phase-contrast microscopy.

### 2.5. Real Time Quantitative PCR (qPCR)

Total and intracellular viral RNAs were prepared for relative qPCR by Trizol reagent (Invitrogen). Then, according to the manufacturer’s instructions of the ReverAid First strand cDNA synthesis kit (Thermo Scientific), 11 μL of viral RNAs and 1.5 μg of total RNAs were reversed transcribed into cDNA. Then 1 μL of cDNA was amplified with forward and reverse primers for EV71 VP1 gene and GAPDH control using LightCycler DNA Master SYBR Green I kit (Roche Diagnostics Corporation, Basel, Switzerland). The forward and reverse EV71 VP1 gene primers were: 5'- GCA GCC CAA AAG AAC TTC AC-3' and 5'- ATT TCA GCA GCT TGG AGT GC-3', respectively. The forward and reverse GAPDH primers were: 5'-TGTTCCAATATGATTCCACCC-3' and 5'- CTTCTCCATGGTGCGTGAAGA-3', respectively. The reactions were performed with the Roche Light Cycler 480 system under the following conditions: Initial denaturation step at 95 °C for 10 min, followed by 40 cycles of 30 sat 94 °C, at 55 °C for 30 s and at 72 °C for 30 s. The CT value was normalized to that of GAPDH. All samples were run in triplicate.

Intracellular EV71 virions for absolute qPCR were prepared as described in Dr. Mingliang He’s paper [30]. A quantitative standard curve was achieved as described in our previous paper [6]. Quantified results were extrapolated from the standard curve with all samples being run in triplicate.

### 2.6. Western Blot Analysis

Western blots were performed as described in our previous study [9]. Cells were harvested at indicated time points and lysed for 1 h in lysis buffer (Santa Cruz) containing complete protease inhibitors (Roche Applied Science). Total protein concentration was determined by the Bicinchoninic Acid Protein Assay Kit (Pierce, Thermo Scientific) after obtaining cell extracts by centrifugation at 13,000 rpm and 4 °C. Before transferred to PVDF membranes (Millipore), proteins were resolved on the sodium dodecyl sulfatesulfate polyacrylamide gel electrophoresis (SDS-PAGE). Then the membranes were blocked in 5% non-fat-dry-milk solution for 1 h at room temperature and then blotted with specific primary antibodies over night at 4 °C, following incubated with horseradish peroxidase antibodies for 1 h at room temperature. The immunoreactive bands were developed with SuperSignal West Femto Maximum Sensitivity Substrate (Pierce, Thermo Scientific) or enhanced chemiluminescent substrate (ECL), followed by autoradiography.

### 2.7. Statistics

All the curves and diagrams were made by using the Graph Pad Prism 5 Program (GraphPad). Data were shown as the mean ± standard deviation (SD) and analyzed by Student’s *t*-test. *p* < 0.05 was considered statistically significant.

## 3. Results

### 3.1. Specific Knockdown of ERK Isoform Did Not Affect the Activation of the Other Isoform Induced by EV71

**Figure 1:**
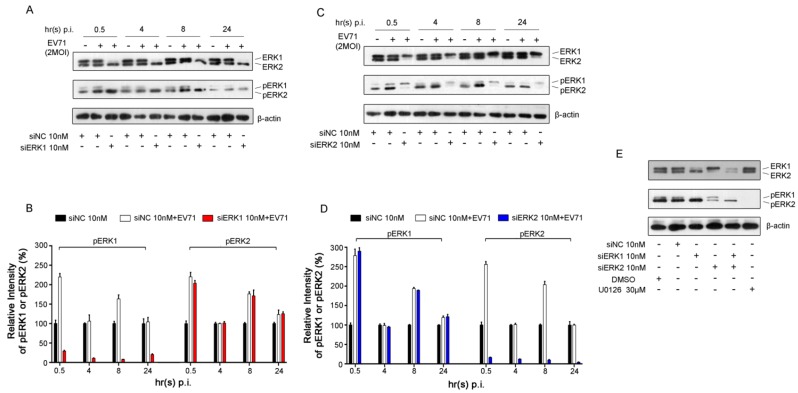
EV71-induced activation of ERK1/2 in Rhabdomyosarcoma (RD) cells. (**A**) RD cells were infected with EV71 (MOI = 2) at 36 h post-transfection with ERK1 siRNA (siERK1). Cells transfected with negative control siRNA (siNC) were used as controls. Cell lysates were collected at the indicated time points post infection (p.i.). ERK1/2, pERK1/2 and β-actin were detected by Western blot analysis, respectively; (**B**) The intensity of pERK1 or pERK2 normalized to that of corresponding ERK was determined by densitometric scanning based on the results from panel A; (**C**) The experiment was performed as described in panel A except that RD cells were transfected with ERK2 siRNA (siERK2); (**D**) Based on the results from panel C, the intensity was determined as described in panel B; (**E**) Cell lysates were collected from RD cells treated with siERK1, siERK2 and both (siERK1+2) at 36 h after transfection and cells treated with U0126 at 12 h after addition. ERK1/2 and pERK1/2 were blotted with specific antibodies. Experiments were repeated three times.

Biphasic activation of ERK1/2 caused by EV71 infection, including early transient and late sustained phases, has been demonstrated in our previous study [6]. To further identify the activation status of specific ERK isoform (ERK1 or ERK2) in viral infection, the phosph-ERK1 (pERK1) and phosph-ERK2 (pERK2) under EV71 infection were determined separately. As shown in Figure 1A and Figure 1B, a biphasic activation of ERK2 induced by EV71 was observed in RD cells pre-treated with ERK1 siRNA (designated as siERK1) compared with that of respective uninfected groups pre-transfected with negative control siRNA (designated as siNC).

No compensatory activation of ERK2 was found when specific knockdown of ERK1. Similarly, the activation of ERK1 was not impacted when specifically silencing of ERK2 with ERK2 siRNA (designated as siERK2) in RD cells infected with EV71 (Figure 1C, D). These data indicated that the activation of ERK1 and ERK2 did not affect one another in the presence of EV71 infection in RD cells.

Notably, siERK1 and siERK2 were confirmed very efficient in knocking down corresponding ERK protein in quiescent RD cells collected 36 h post transfection and detected by Western blot analysis, which correlated with a strong decrease in ERK phosphorylation (Figure 1E). Moreover, treatment of U0126, a specific inhibitor of ERK activation, decreased more than 99% of ERK1/2 activation at 12 h after addition. No significant cytotoxicity of siERK1, siERK2, siERK1+2 and U0126 to the proliferation and survival of RD cells were observed in the current study (Figure S1).

### 3.2. Depletion of Individual ERK Isoform Resulted in a Similar Reduction of EV71 Proliferation

**Figure 2:**
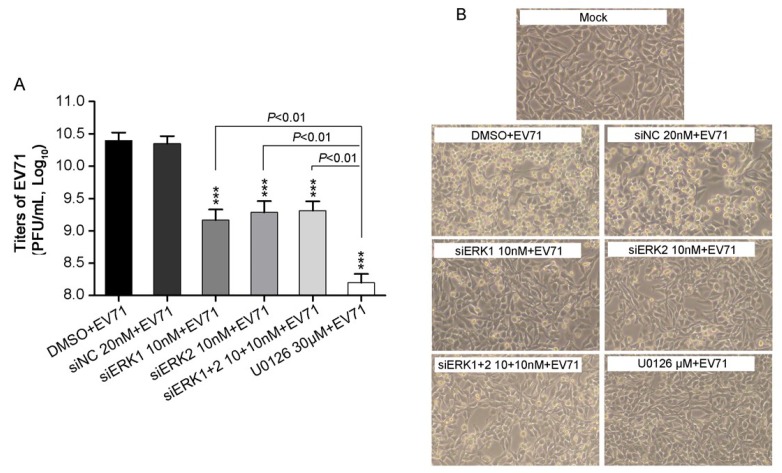
EV71 titers and CPE in RD cells treatment with distinct siERK (s) or U0126. (**A**) RD cells, pre-transfected with siERK1, siERK2 or siERK1+2 for 36 h, respectively, or treated with U0126 1 h prior to infection, were infected with EV71 at an MOI of 2. Cells pre-treated with DMSO or siNC were done as parallels. At 24 h p.i., both supernatants and cell lysates were collected and applied for determining the total titers of EV71 by titration; (**B**) Experiments were performed as described in panel A except CPE was examined every 8 h and images were taken at 24 h p.i.. Each result represents the average of three independent experiments and is shown as the means ± standard deviations (SD). *** *p* < 0.001, *versus* corresponding controls by Student’s *t*-test.

It has been demonstrated that MEK1 and MEK2 play a different role in EV71 replication [6]. To further elucidate the roles of downstream kinases of MEKs, ERK1 and ERK2 in EV71 replication, the effects of distinct knockdown of ERK1 or ERK2, or both on progeny viral titers were investigated. A clear reduction of progeny EV71 titers by about 90% were obtained in cells pre-treated with siERK1, siERK2 and siERK1+2, respectively, when compared with that of siNC control (Figure 2A).

No obvious difference of progeny viral titers was observed either knocking down of both ERK1 and ERK2, or each, whereas a stronger reduction of progeny viral titers was found when treating cells with U0126. In addition, EV71-induced cytopathic effect (CPE) was examined under phase-contrast microscopy. At 24 h p.i., pre-treatment with siERK1, siERK2, siERK1+2 or U0126 remarkably suppressed the morphological changes caused by EV71 infection (Figure 2B), as compared to corresponding controls, which was consistent with the viral titers in corresponding groups. Taken together, these results indicated ERK1 and ERK2 were required, but not functionally redundant for EV71 proliferation.

### 3.3. Distinct Knockdown of ERK Isoform Reduced Viral Genomic RNAs

**Figure 3:**
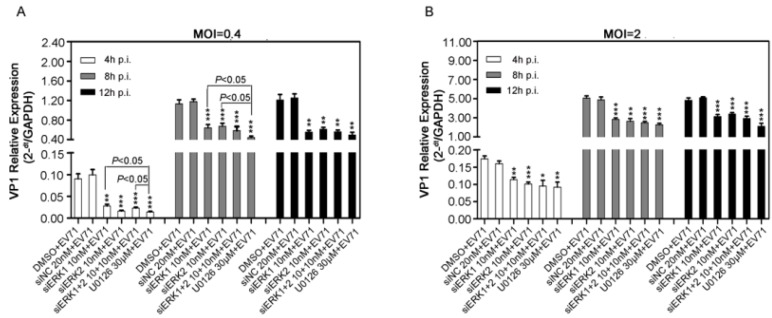
Effects of ERK1 and ERK2 on viral RNA synthesis in RD cells. RD cells pre-treated with siERK(s) or U0126 were infected with EV71 at the MOI of 0.4 (panel **A**) or 2 (panel **B**). Then at the indicated time points p.i., both supernatants and cell lysates were collected, viral VP1 gene among total viral genomes was quantified by relative qPCR. Data were the means of three independent experiments and error bars were denoted the SD. ** *p* < 0.01 *** *p* < 0.001, *versus* respective controls by Student’s *t-*test.

To further identify the effects of ERK1 and ERK2 on EV71 replication cycle, VP1 gene among total viral RNAs extracted from siERK(s)- and U0126-treated RD cells was quantified by relative quantitative PCR (qPCR). Viral VP1 gene was significantly suppressed by about 30% to 40% at 4 h, 8 h and 12 h p.i. by siERK1, siERK2, siERK1+2 and U0126 when compared with corresponding controls in RD cells infected with EV71 (Figure 3).

In addition, U0126 caused a stronger reduction of viral genomic RNA than those of siERK1 and siERK1+2 at 4 and 8 h p.i., when RD cells was infected with 0.4 MOI of EV71 (Figure 3A). It should be noted that amounts of VP1 gene remained almost unchanged in each group from 8 h p.i. to 12 h p.i., indicating that the life cycle of EV71 might be less than 8 h. All these results revealed that both ERK1 and ERK2 might play important roles in viral RNA synthesis.

### 3.4. Disruption of Either ERK1 or ERK2 Resulted in a Reduction of EV71 Protein

**Figure 4:**
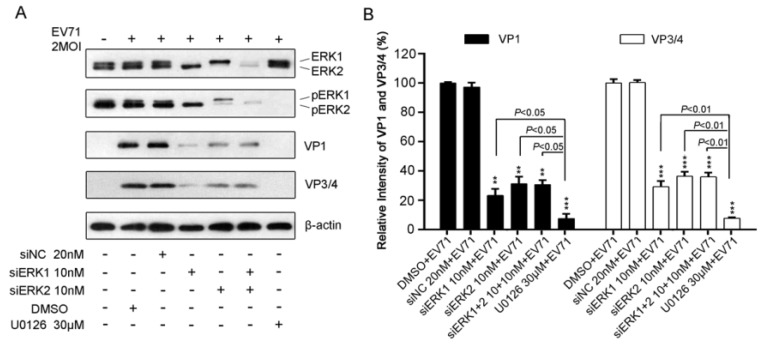
Impacts of ERK1 and ERK2 on protein production of EV71 in RD cells. (**A**). RD cells pre-treated with siERK(s) or U0126 were infected with 2 MOI of EV71. At 12 h p.i., total protein harvested was blotted with antibodies specific to VP1 and VP3/4. β-actin was used as the loading control. The relative intensities of VP1 (panel **B**) and VP3/4 (panel **C**) normalized to β-actin were presented by the percentage of the intensity of respective DMSO+EV71 group (100%). Data represent the average of three independent experiments and is shown as the means ± SD. ** *p* < 0.01 *** *p* < 0.001, *versus* corresponding control groups by Student’s *t*-test.

To further determine the impact of specific ERK isoform on EV71 protein, viral structural proteins VP1 and VP3/4 were detected by Western blot analysis in EV71-infected RD cells pre-transfected with siERK1,or siERK2, or both, respectively. As shown in Figure 4A, ERK1, or ERK2, or ERK1/2 expression were almost diminished by siERK1, or ERK2, or siERK1+2, which resulted in the activation inhibition of ERK1, ERK2, or ERK1/2 by about 80% to 90%. The silencing effect decreased the expression of VP1 and VP3/4 by around 70%, when compared with that of siNC control (Figure 4B).

It seemed that siERK1 had a better inhibitory effect on VP1 and VP3/4 but no significance difference compared with that of siERK2 and siERK1+2 (Figure 4B). In addition, U0126 reduced more than 99% of viral VP1 and VP3/4 expression which was significantly reduction than that of siERK(s)-treated groups. Our results suggested that both ERK1 and ERK2 might be crucial for EV71 protein production.

### 3.5. Inhibiting both Early and Late Phases of ERK1/2 Activation Showed No Significant Difference from Blocking only the Late One for EV71 Replication 

Since depletion of ERK1 or ERK2 both impaired virus replication severely, a time-of-drug addition assay was performed next in EV71-infected RD cells at the indicated time points to further specify the roles of the two phases of ERK1/2 activation in the viral life cycle. VP1 gene representing intracellular viral RNAs or intracellular virions was determined.

**Figure 5:**
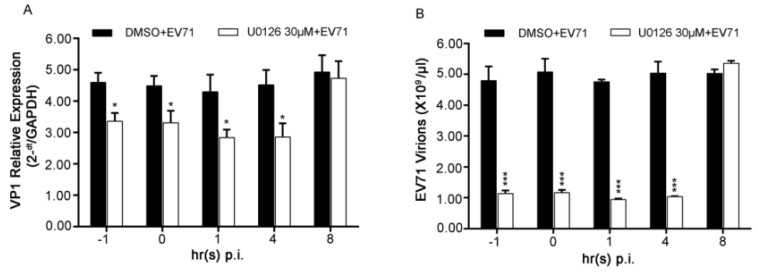
Effects of early ERK1/2 activation on EV71 infection in RD cells. (**A**) RD cells infected with EV71 at the MOI of 2 was treated with 30 μM of U0126 at the indicated time points p.i.. At 12 h p.i., cells were collected and viral VP1 gene among intracellular viral RNAs was quantified by relative qPCR; (**B**) The experiments were performed as described in panel A except 100 μg/mL of RNase A was used to eliminate naked viral RNAs before RNA was extracted. VP1 gene among intracellular EV71 virions was quantified by absolute qPCR. Data shown were the means ± SD (*n* = 3). * *p* < 0.05 ** *p* < 0.01 *** *p* < 0.001, *versus* respective controls by Student’s *t*-test.

As shown in Figure 5A, disturbing activation of both ERK1 and ERK2 by treating cells with U0126 at 1 h before infection (−1), 0 h (0), 1 h(1) and 4 h (4) p.i. resulted in about 30% of suppression of intercellular viral RNAs. The similar results of intercellular virions were also obtained in the time-of-drug addition assay (Figure 5B). These data suggested that the late phase of ERK1/2 activated by EV71 might be more important for EV71 life cycle which may include viral RNA replication and/or translation.

## 4. Discussion

Viruses hijack components of host’s metabolic machinery for productive replication. The significance of activation of ERK pathway, as one of the specific responses to infection of varieties of viruses including EV71 [5,6,7,8,9,10,11,31,32], has been broadly reported. Our previous study has established the key role of MEK1 in the activation of ERK pathway induced by EV71 and proposed that MEK1-ERK1/2 signaling pathway acts as a central “hub” in EV71 replication [6]. However, it is still unclear if ERK1 and ERK2, the only known downstream kinases of MEK1 to date, play distinct role(s) in the life cycle of EV71. In fact, there are many studies focusing on distinction of ERK1 and ERK2 in several other areas rather than in virus replication. Meanwhile, the conclusions of these studies yet remain controversial. Although ERK1 and ERK2 are inclined to be thought of interchangeably [23], increasing studies have provided evidences for differential roles of ERK1 and ERK2 in cell movement [24], embryonic mice viability [33,34,35], and pathophysiology of central neural system *etc.* [36]. Our present work investigated distinction(s) of the individual involvement of ERK1 and ERK2 in EV71 replication.

Consistent with previous studies [6], EV71 infection caused a non-discriminative biphasic activation of ERK1/2 in RD cells. Some literatures reported that depletion of ERK1 induced higher activation of ERK2 in MEFs [34] and removal of ERK2 led to increased activation of ERK1 in NIH3T3 cells [37]. However, in our study, single silencing specific ERK isoform did not lead any significant effect on the biphasic activation of the other isoform in cells infected with or without EV71 (Figure 1), indicating that the activation of ERK1 and ERK2 might be various under different circumstances, although the mechanism is elusive.

Moreover, the present study shows both ERK1 and ERK2 are essential and not functionally redundant for EV71 replication due to the fact that individually silencing specific ERK isoform impaired EV71 propagation significantly, resulting in a marked suppression of viral progeny titer and EV71-induced CPE (Figure 2) as well as a clear reduction of viral RNA and protein synthesis (Figure 3 and Figure 4). U0126 showed stronger inhibitory effect on virus replication than ERK siRNA(s) did, probably because U0126 was able to block the activation of ERK pathway more efficiently. Interestingly, double knockdown of ERK1/2 by siRNAs suppressed virus propagation, viral RNAs and proteins to a similar level compared with those of single knockdown groups, indicating that ERK1 and ERK2 probably work together in EV71 life cycle in RD cells. In addition, it is reasonable to hypothesize that activated ERK1 and ERK2 act as a functional ensemble rather than playing their roles separately in EV71 replication. This is the first to report that ERK1 and ERK2 are both required as key factors in EV71 efficient propagation, which is quite different from distinctive or interchangeable functions of ERK1 and ERK2 in several other areas [21,22,23,24,33,34]. Combined with our previous study on MEK1 [6], the upstream kinase of ERK1/2, these findings further verified the critical role of MEK1-ERK1/2 pathway in EV71 life cycle.

In addition, according to the time-of-drug addition assay (Figure 5), it appeared that virus successful biosynthesis process was more closely related to the late phase of ERK1/2 activation induced by EV71 than the early one. This result was consistent with and further confirmed previous studies in which the early phase of ERK1/2 activation is not involved in viral propagation directly whereas might be the consequence of the virus-receptor binding [6,11].

In conclusion, this study provided evidence that ERK1 and ERK2 are not interchangeable in the EV71 life cycle and indicated they might act their functions as a whole. The findings deepened our understanding in the roles played by MEK1-ERK1/2 signaling pathway in virus life cycle and shed light on possibilities to offer flexible choices to selectively target the one between ERK1 and ERK2 in future anti-viral strategies. Further studies will be needed to specify the effect(s) and mechanism(s) of ERK1/2 on specific EV71 replication process(es).

## 5. Conclusions

Overall, the present study identified the effects of specific ERK isoform on EV71 propagation as well as on the viral genomic RNAs and proteins. Our data revealed that ERK1 and ERK2 are both required and not interchangeable for EV71 life cycle. The activations of ERK1 and ERK2 induced by EV71 were non-discriminative and independent to each another. Besides, the late phase of ERK1/2 activation under infection might be more responsible for the replication of EV71.

## Supplementary Files

Supplementary File 1

## References

[1] The Picornavirus Pages: Enterovirus A. http://www.picornaviridae.com/enterovirus/ev-a/ev-a.htm.

[2] Lin J.Y., Chen T.C., Weng K.F., Chang S.C., Chen L.L., Shih S.R. (2009). Viral and host proteins involved in picornavirus life cycle. J. Biomed. Sci..

[3] Yi L., Lu J., Kung H.F., He M.L. (2011). The virology and developments toward control of human enterovirus 71. Crit. Rev. Microbiol..

[4] Wang S.M., Liu C.C. (2014). Update of enterovirus 71 infection: epidemiology, pathogenesis and vaccine. Expert Rev. Anti Infect. Ther..

[5] Tung W.H., Hsieh H.L., Lee I.T., Yang C.M. (2011). Enterovirus 71 modulates a COX-2/PGE2/cAMP-dependent viral replication in human neuroblastoma cells: role of the c-Src/EGFR/p42/p44 MAPK/CREB signaling pathway. J. Cell Biochem..

[6] Wang B., Zhang H., Zhu M., Luo Z., Peng Y. (2012). MEK1-ERKs signal cascade is required for the replication of Enterovirus 71 (EV71). Antivir. Res..

[7] Cai Y., Liu Y., Zhang X. (2007). Suppression of coronavirus replication by inhibition of the MEK signaling pathway. J. Virol..

[8] Pleschka S., Wolff T., Ehrhardt C., Hobom G., Planz O., Rapp U.R., Ludwig S. (2001). Influenza virus propagation is impaired by inhibition of the Raf/MEK/ERK signalling cascade. Nat. Cell Biol..

[9] Zhang H., Feng H., Luo L., Zhou Q., Luo Z., Peng Y. (2010). Distinct effects of knocking down MEK1 and MEK2 on replication of herpes simplex virus type 2. Virus Res..

[10] Panteva M., Korkaya H., Jameel S. (2003). Hepatitis viruses and the MAPK pathway: Is this a survival strategy?. Virus Res..

[11] Luo H., Yanagawa B., Zhang J., Luo Z., Zhang M., Esfandiarei M., Carthy C., Wilson J.E., Yang D., McManus B.M. (2002). Coxsackievirus B3 replication is reduced by inhibition of the extracellular signal-regulated kinase (ERK) signaling pathway. J. Virol..

[12] Murphy L.O., Blenis J. (2006). MAPK signal specificity: The right place at the right time. Trends Biochem. Sci..

[13] McCubrey J.A., Steelman L.S., Chappell W.H., Abrams S.L., Wong E.W., Chang F., Lehmann B., Terrian D.M., Milella M., Tafuri A. (2007). Roles of the Raf/MEK/ERK pathway in cell growth, malignant transformation and drug resistance. Biochim. Biophys. Acta.

[14] Zhang W., Liu H.T. (2002). MAPK signal pathways in the regulation of cell proliferation in mammalian cells. Cell Res..

[15] Kim E.K., Choi E.J. (2010). Pathological roles of MAPK signaling pathways in human diseases. Biochim. Biophys. Acta.

[16] Deschenes-Simard X., Kottakis F., Meloche S., Ferbeyre G. (2014). ERKs in cancer: Friends or foes?. Cancer Res..

[17] Arthur J.S., Ley S.C. (2013). Mitogen-activated protein kinases in innate immunity. Nat. Rev. Immunol..

[18] Tanti J.F., Jager J. (2009). Cellular mechanisms of insulin resistance: Role of stress-regulated serine kinases and insulin receptor substrates (IRS) serine phosphorylation. Curr. Opin. Pharmacol..

[19] Chambard J.C., Lefloch R., Pouyssegur J., Lenormand P. (2007). ERK implication in cell cycle regulation. Biochim. Biophys. Acta.

[20] Boulton T.G., Nye S.H., Robbins D.J., Ip N.Y., Radziejewska E., Morgenbesser S.D., DePinho R.A., Panayotatos N., Cobb M.H., Yancopoulos G.D. (1991). ERKs: A family of protein-serine/threonine kinases that are activated and tyrosine phosphorylated in response to insulin and NGF. Cell.

[21] Roskoski R. (2012). ERK1/2 MAP kinases: structure, function, and regulation. Pharmacol. Res..

[22] Lefloch R., Pouyssegur J., Lenormand P. (2009). Total ERK1/2 activity regulates cell proliferation. Cell Cycle.

[23] Yoon S., Seger R. (2006). The extracellular signal-regulated kinase: Multiple substrates regulate diverse cellular functions. Growth Factors.

[24] Krens S.F., He S., Lamers G.E., Meijer A.H., Bakkers J., Schmidt T., Spaink H.P., Snaar-Jagalska B.E. (2008). Distinct functions for ERK1 and ERK2 in cell migration processes during zebrafish gastrulation. Dev. Biol..

[25] Vantaggiato C., Formentini I., Bondanza A., Bonini C., Naldini L., Brambilla R. (2006). ERK1 and ERK2 mitogen-activated protein kinases affect Ras-dependent cell signaling differentially. J. Biol..

[26] Lloyd A.C. (2006). Distinct functions for ERKs?. J. Biol..

[27] Cargnello M., Roux P.P. (2011). Activation and function of the MAPKs and their substrates, the MAPK-activated protein kinases. Microbiol. Mol. Biol. Rev..

[28] Wang B., Ding L.X., Deng J., Zhang H., Zhu M., Yi T., Liu J., Xu P., Lu F.M., Peng Y.H. (2010). Replication of EV71 was suppressed by MEK1/2 inhibitor U0126. Chin. J. Biochem. Mol. Biol..

[29] Dimitri C.A., Dowdle W., MacKeigan J.P., Blenis J., Murphy L.O. (2005). Spatially separate docking sites on ERK2 regulate distinct signaling events *in vivo*. Curr. Biol..

[30] Lu J., He Y.Q., Yi L.N., Zan H., Kung H.F., He M.L. (2011). Viral kinetics of enterovirus 71 in human abdomyosarcoma cells. World J. Gastroenterol..

[31] Rodriguez M.E., Brunetti J.E., Wachsman M.B., Scolaro L.A., Castilla V. (2014). Raf/MEK/ERK pathway activation is required for Junin virus replication. J. Gen. Virol..

[32] Pleschka S. (2008). RNA viruses and the mitogenic Raf/MEK/ERK signal transduction cascade. Biol. Chem..

[33] Yao Y., Li W., Wu J., Germann U.A., Su M.S., Kuida K., Boucher D.M. (2003). Extracellular signal-regulated kinase 2 is necessary for mesoderm differentiation. Proc. Natl. Acad. Sci. USA.

[34] Pages G., Guerin S., Grall D., Bonino F., Smith A., Anjuere F., Auberger P., Pouyssegur J. (1999). Defective thymocyte maturation in p44 MAP kinase (Erk 1) knockout mice. Science.

[35] Nekrasova T., Shive C., Gao Y., Kawamura K., Guardia R., Landreth G., Forsthuber T.G. (2005). ERK1-deficient mice show normal T cell effector function and are highly susceptible to experimental autoimmune encephalomyelitis. J. Immunol..

[36] Yu C. (2012). Distinct roles for ERK1 and ERK2 in pathophysiology of CNS. Front. Biol..

[37] Lefloch R., Pouyssegur J., Lenormand P. (2008). Single and combined silencing of ERK1 and ERK2 reveals their positive contribution to growth signaling depending on their expression levels. Mol. Cell Biol..

