# Supplementary material for: Both ERK1 and ERK2 Are Required for Enterovirus 71 (EV71) Efficient Replication
Source: Viruses. 2015 Mar 20;7(3):1344–56. doi: 10.3390/v7031344 (PMC4379574; doi:10.3390/v7031344)
Supplement: Supplementary File 1 [file viruses-07-01344-s001.pdf]

Supplementary Material

To rule out the possibility that it may cause adverse effects on RD cells when inhibiting ERK pathway by siERK(s) or U0126, LDH assay and cell counting were performed to check the cell survival and proliferation, respectively. As shown in Figure S1A, the survival rate (obtained from LDH assay) of RD cells treated with siERK(s) showed no significant difference from that of respective mock transfected group at either 48 h or 96 h post transfection.

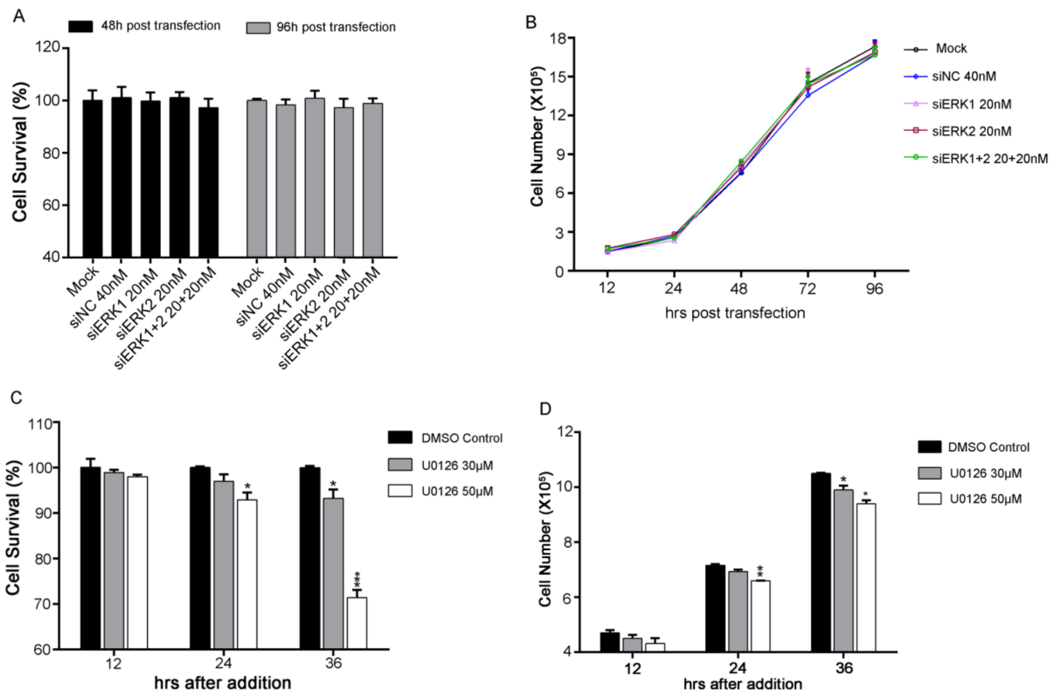

Figure S1. Effects of siERK(s) and U0126 on the survival and growth of RD cells. *(A)*. 12h after 1 x 10^5 of RD cells were seeded, cells were transfected with siERK1, or siERK2, or siERK1+2 at the indicated concentration. LDH assay was applied at the indicated time points post transfection. The survival rates of siERK(s) treated groups were normalized to that of respective Mock transfected group (100%). *(B)*. Experiments were carried out as described in panel A except cell numbers were counted at the indicated time points post transfection. *(C)*. At 12h after 3 x 10^5 of cells were planted, RD cells were treated with U0126 at the concentrations of 30 μM and 50 μM. Then LDH assay was done at the indicated time points after the addition of U0126. The survival rates of U0126 treated groups were normalized to that of respective DMSO treated group (100%). *(D)*. Experiments were carried out as described in panel C except cell numbers were counted at the indicated time points after U0126 addition. Data shown were means ± SD (n = 3). ***P*<0.01 ****P*<0.001, versus corresponding controls by Student's *t*-test.

Meanwhile, no any remarkable effect on cell proliferation was found in cells treated with siERK(s) (Figure S1B). However, a markedly reduction in survival rates and cell numbers was observed in cells exposed to 30 μM of U0126 at 36 h after addition and 50 μM of U1026 at both 24 h and 36 h after addition, compared with corresponding control groups (Figure S1C and S1D). All the results showed that under the experimental conditions in the present study, siERK(s) and U0126 would not interfere with the survival and proliferation of RD cells.
